# Sepsis causes neuroinflammation and concomitant decrease of cerebral metabolism

**DOI:** 10.1186/1742-2094-5-38

**Published:** 2008-09-15

**Authors:** Alexander Semmler, Sven Hermann, Florian Mormann, Marc Weberpals, Stephan A Paxian, Thorsten Okulla, Michael Schäfers, Markus P Kummer, Thomas Klockgether, Michael T Heneka

**Affiliations:** 1 University Bonn, Department of Neurology, Bonn, Germany; 2 University Münster, Department of Nuclear Medicine, Münster, Germany; 3 University Bonn, Department of Epileptology, Bonn, Germany

## Abstract

**Background:**

Septic encephalopathy is a severe brain dysfunction caused by systemic inflammation in the absence of direct brain infection. Changes in cerebral blood flow, release of inflammatory molecules and metabolic alterations contribute to neuronal dysfunction and cell death.

**Methods:**

To investigate the relation of electrophysiological, metabolic and morphological changes caused by SE, we simultaneously assessed systemic circulation, regional cerebral blood flow and cortical electroencephalography in rats exposed to bacterial lipopolysaccharide. Additionally, cerebral glucose uptake, astro- and microglial activation as well as changes of inflammatory gene transcription were examined by small animal PET using [18F]FDG, immunohistochemistry, and real time PCR.

**Results:**

While the systemic hemodynamic did not change significantly, regional cerebral blood flow was decreased in the cortex paralleled by a decrease of alpha activity of the electroencephalography. Cerebral glucose uptake was reduced in all analyzed neocortical areas, but preserved in the caudate nucleus, the hippocampus and the thalamus. Sepsis enhanced the transcription of several pro- and anti-inflammatory cytokines and chemokines including tumor necrosis factor alpha, interleukin-1 beta, transforming growth factor beta, and monocot chemoattractant protein 1 in the cerebrum. Regional analysis of different brain regions revealed an increase in ED1-positive microglia in the cortex, while total and neuronal cell counts decreased in the cortex and the hippocampus.

**Conclusion:**

Together, the present study highlights the complexity of sepsis induced early impairment of neuronal metabolism and activity. Since our model uses techniques that determine parameters relevant to the clinical setting, it might be a useful tool to develop brain specific therapeutic strategies for human septic encephalopathy.

## Background

Sepsis and its complications are the leading causes of mortality in intensive care units accounting for 10–50% of deaths. Up to 71% of septic patients develop potentially irreversible acute cerebral dysfunction [1-3]. This sepsis-induced encephalopathy is caused by systemic inflammation in the absence of direct brain infection and clinically characterized by slowing of mental processes, impaired attention, disorientation, delirium or coma. Importantly, septic encephalopathy (SE) is an early sign of sepsis and associated with an increased rate of morbidity and mortality [2].

The pathogenesis of SE is unlikely to be directly induced by a pathogenic toxin, as similar encephalopathy can develop as a result of a number of systemic inflammatory response syndromes that lack an infectious etiology (e.g. acute pancreatitis, burns etc.). Clinical and experimental data suggest that a number of factors including the local generation of pro-inflammatory cytokines, impaired cerebral microcirculation, an imbalance of neurotransmitters and a negative impact of peripheral organ failure contribute to the development of SE. Additionally, once inflammation persists, increased excitotoxicity and oxidative stress may further aggravate SE and contribute to neuronal dysfunction and degeneration (for review see [3]). Of note, patients with a pre-existing CNS pathology have a higher risk to develop SE, and a similar predisposing interaction has been reported in an animal model of sepsis [4].

Clinically, the electroencephalogram (EEG) serves as an important diagnostic tool for SE assessment and the majority of patients shows abnormal EEG recordings [5]. Of note, the degree of EEG pathology correlates well with the clinical status and prognosis and has been proven more sensitive than clinical bedside investigation [5]. Likewise, cerebral blood flow (CBF) is another parameter which is routinely analyzed in patients suffering from SE, based on the assumption that sepsis exerts profound and sustained effects on the systemic circulatory function. However, past studies have yielded controversial results and to date, the effects of sepsis on CBF as well as neuronal metabolism and activity remain unclear. To further investigate the relation of potential regional CBF changes, electroencephalography and cerebral metabolism in response to SE, we investigated hemodynamic, electrophysiological and metabolic changes in relation to neuroinflammatory markers and neuronal number in a model of acute SE in rats. Regional cerebral blood flow was reduced in correlation to EEG frequency 24 h after intraperitoneal injection of LPS, whereas brain glucose utilization and neuronal number were reduced concurrent with microgliosis and neuroinflammatory response.

## Methods

### Animals

53 male Wistar rats (Charles River, Sulzfeld, Germany) weighing 250 – 300 g were housed in groups under standard conditions at a temperature of 22°C (± 1°C) and a 12 hour light-dark cycle – with free access to standard food (Altromin, Soest, Germany) and tap water. Animal care and handling were performed according to the Declaration of Helsinki and approved by local ethical committees (approval number 50.203.2 BN 33,34/00).

Rats were randomized and received either 10 mg/kg of LPS (0127:B8, E. coli; Sigma, München, Germany) dissolved in 1 ml sodium chloride (0.9%) intraperitoneally (i. p.) or the vehicle alone. 24 hours after induction of sepsis, animals were anaesthetized with a combination of ketamine (80 mg/kg) and xylazine (10 mg/kg). The trachea was cannulated to facilitate respiration and rectal temperature was maintained at 37°C using a heating blanket. The femoral artery was exposed and catheterized with a polyethylene tube connected to a pressure transducer for continuous recording of arterial blood pressure and heart rate (Harvard Apparatus, March-Hugstetten, Germany). The head was fixed in a stereotactic frame and four stainless steel skull screws were placed epidurally, two electrodes per parietal bone at bregma coordinates 0 mm and -6.5 mm, 4 mm from the midline. A reference electrode was placed on the anterior midline over the frontal sinus. All electrodes were connected through insulated wire with a 2-channel amplifier (Harvard Apparatus, March-Hugstetten, Germany). Electrical brain activity was amplified (× 10 000 – 20 000), digitized and transferred to a PC for storage and further analysis. EEG was recorded for 15 – 20 min periods.

After EEG-recording, the screws were removed, and a 5 × 3 mm large cranial window was drilled (thinning of the scull until translucency, leaving the dura mater intact), centered 4 mm lateral and 4 mm caudal to the bregma. CBF was assessed using a laser flow blood perfusion monitor (PeriFlux 5000, Perimed, Stockholm, Sweden) with a 1.0 mm diameter laser Doppler probe (wavelength 780 μm; probe 407, Perimed, Stockholm Sweden). Local CBF was measured sequentially at 32 (8 × 4) parietocortical sites 300 μm apart, just above the dura over the exposed hemisphere using a micromanipulator as previously published [6]. Data were sampled, on average, for 8 s at each site. Intraarterial pressure curve and CBF signals were transferred to a PC (Haemodyn, Harvard Apparatus, March-Hugstetten, Germany). Signals were sampled at 500 Hz with a 12 bit resolution. All experiments were carried out in accordance with the animal welfare guidelines and laws of the Federal Republic of Germany and were approved by the local ethics committee.

### Small animal positron emission tomography (microPET)

microPET was performed on a 32-module quadHIDAC scanner (Oxford Positron Systems, Weston-on-the-Green, UK) dedicated to small animal imaging. The scanner has an effective resolution of 0.7 mm (FWHM) in the transaxial and axial directions when using an iterative resolution recovery reconstruction algorithm [7].

Before the scan, a tail vein catheter was inserted under short-term isofluorane anesthesia. The conscious rat was afterwards kept in a restraining device. 40 MBq of ^18^F-Fluordeoxyglucose (FDG) in 800 μl 0.9% saline were injected via the tail vein catheter. Following a 60 min interval, animals were again anaesthetized using isofluorane and placed in the PET scanner on a heating pad to maintain normal body temperature. List mode PET data were acquired for 15 minutes and subsequently reconstructed into a single image volume with a voxel size of 0.8 × 0.8 × 0.8 mm^3^.

### Immunohistochemistry

Rats were perfused transcardially using heparanized saline and brains were subsequently dissected. Serial sagittal sections were cut (10 μm) from cryo-conserved preserved hemispheres (Leica Cryostat CM 3050S), embedded in tissue freezing medium (Leica Microsystems #0201-08926, Nussloch, Germany) and mounted (Microscope Slides #K0123b, Engelbrecht, Germany). After drying for 30 minutes at room temperature, for fixation slides were incubated in 4% paraformaldehyde (Roti Histofix 4% #P087.4, Roth, Karlsruhe, Germany) for 20 minutes. Blocking of non-specific binding was achieved by one hour incubation in 5% normal goat serum (Linaris #S-1000, Wertheim, Germany). Between the steps, slides were rinsed three times for five minutes in PBST. Immunostaining was performed overnight by incubation at 4°C with the following primary antibodies: 1.) polyclonal antibody rabbit-anti-mouse GFAP (1:1000 in 2% normal goat serum in PBST; DAKO Z0334, Glostrup, Denmark). 2.) monoclonal antibody mouse-anti-rat CD68/ED1 (1:100 in 2% normal goat serum in PBST; Serotec MCA341G, Düsseldorf, Germany). 3.) monoclonal rabbit anti mouse NeuN (1:250 in 2% normal donkey serum in PBS; Clone A60, Chemicon, Temecula, CA). Afterwards slides were incubated with Alexa Fluor 594-labeled secondary antibodies for one hour (1:400 in PBST; Invitrogen #A11037 & #A11020 Karlsruhe, Germany). For co-staining with Hoechst Dye 33342 (10 μg/ml; Fluka/Sigma-Aldrich #14533, Steinheim, Germany) an incubation time of two minutes was set. Again, slides were rinsed with PBST between the steps. Finally, the slides were covered in Mowiol 4–88 (Calbiochem/VWR #475904, Darmstadt, Germany) and stored at -20°C in the dark until microscopy was performed.

### Real time PCR

RNA from brain hemispheres were extracted using Trizol (Life Technologies Invitrogen, Karlsruhe, Germany) using an Ultra Turrax (IKA Labortechnik, Staufen, Germany). Total RNA was quantified photometrically and reverse transcribed using the RevertAid First Strand cDNA Synthesis kit (Fermentas, St. Leon-Rot, Germany) according to the manufacturer's instructions. Real time qPCR was performed using the StepOnePlus™ Real-Time PCR System (Applied Biosystems, Foster City, USA). Power SYBR^® ^Green PCR Master Mix (Applied Biosystems, Foster City, USA) was used for PCR amplification and real time detection of PCR products. 1 μl of the RT product corresponding to 40 ng of total RNA, 0.2 μM of each primer and 10 μl of the master mix were mixed and run under the following conditions: 95°C for 10 min and 40 cycles of 95°C for 15 s and 60°C for 1 min. Amplification specificity was checked using a melting curve analysis after PCR. mRNA expression was normalized to GAPDH. Primers used were: GAPDH forward ACG ACA GTC CAT GCC ATC AC and reverse TCC ACC ACC CTG TTG CTG TA, IL-1β forward GCT ACC TAT GTC TTG CCC GTG GAG and revers GTC CCG ACC ATT GCT GTT TCC TA, IL-4 forward GGA TGT AACGAC AGC CCT C and revers GAC ACC TCT ACA GAG TTT CC, IL-6 forward CTT GGG ACT GAT GTT GTT GA and revers CTC TGA ATG ACT CTG GCT TTG, IL-10 forward CCT GCT CTT ACT GGC TGG AG and reverse CTG CAG TAA GGA ATC TGT CAG, TNF-α forward AAA ACT CGA GTG ACA AGC CC and reverse GGT TGA CCT CAG CGC TGA GC, TGF-β forward TGC GCC TGC AGA GAT TCA AG and reverse TCT CTG TGG AGC TGA AGC AG, MCP-1 forward CTG TTG TTC ACA GTT GCT GC and revers CTG ATC TCA CTT GGT TCT GG and iNOS forward CCA GAG CAG TAC AAG CTC AC and revers CCA CAA CTC GCT CCA AGA TC.

### Data analysis

Electrophysiological recordings were analyzed using a moving window analysis with a window length of 16.384 s (8192 data points). Prior to analysis signals were scanned for movement and recording artifacts using automated artifact detection. Windows containing constant signals for more than 100 consecutive points or signal jumps exceeding 10 standard deviations of a window's amplitude distribution were discarded.

Arterial pressure signals were decomposed into their instantaneous frequency and envelope signals using the Hilbert transform [8] in order to extract heart rate as well as systolic and diastolic arterial pressure. The shock index (SI) was determined from the ratio of the heart rate to systolic blood pressure. Doppler flow signals were averaged over time. EEG signals were subjected to spectral analysis to extract signal power in different frequency bands using conventional band definitions (delta 0.5–4 Hz, theta 4–8 Hz, alpha 8–13 Hz, beta 13–20 Hz, gamma 20–40 Hz). In order to quantify electroencephalographic signs of septic encephalopathy in terms of slowing of oscillatory brain activity, we determined the main EEG frequency from the maximum of the power spectrum.

All electrophysiological variables extracted in the moving window analysis were averaged over time (i.e., over different windows) for each animal. Results from different groups (septic animals vs. control animals) were compared using a nonparametric test (two-sided Mann-Whitney test for independent samples). In addition, to address the question whether electroencephalographic signs of encephalopathy exhibit dependence on hemodynamic sepsis parameters, we tested for significant correlation using a one-sided non-parametric correlation test (Spearman's rho).

For analyses of FDG-PET the individual volume data sets of all ten PET scans were coregistered and an averaged data set of the control group was calculated on pixel-by-pixel basis using the MPI-Tool (Advanced Tomo Vision, Kerpen, Germany). On this average image regions of interest (ROI) encompassing neocortex, caudate nucleus, thalamus and hippocampus were defined. ROIs were projected onto the individual data sets of all animals in both study groups to assess regional FDG uptake. Cerebral glucose uptake was finally quantified as the count ratio of individual ROIs and a reference ROI placed in the cerebellum in each animal.

For immunofluorescence quantification, 5 randomly chosen areas from 10 parallel sections per animal were analyzed using an Olympus BX61 microscope (Olympus, Hamburg, Germany) at 10× magnification. Evaluation was performed by determining the stained area using Cell^P software (Olympus Soft Imaging Solutions, Münster, Germany). Statistical analysis was performed using the Prism 4 Software, (GraphPad, San Diego, CA).

## Results

### LPS-induced decrease in cortical CBF and alpha activity in EEG

Induction of sepsis by intraperitoneal injection of LPS led to typical sickness behavior (e.g. piloerection, tachypnoe, social withdrawal) of the mice within 2 h. Five out of 27 animals died within the 24 h observation period after LPS administration.

**Figure 1:**
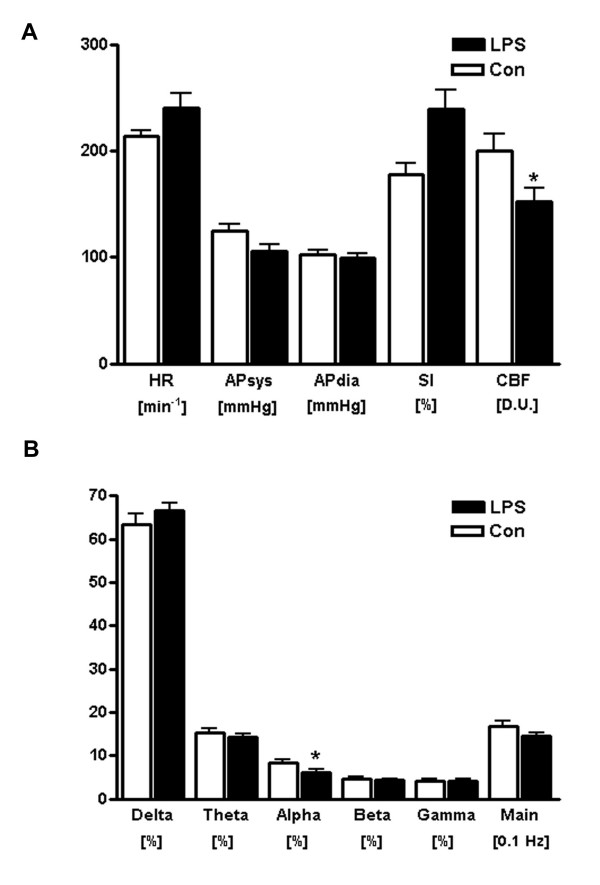
**Changes of hemodynamics and electroencephalography in response to sepsis induction Sample figure title**. A, Displayed are heart rate (HR), systolic and diastolic arterial pressure (APsys, APdia), shock index (SI) and cerebral blood flow (CBF) in vehicle-treated (Con) and LPS-treated rats at 24 h after sepsis induction. While the general cardiovascular parameters did not change significantly, CBF was markedly reduced upon LPS exposure. B, Shown is the relative spectral band power and the main spectral frequency determined from EEG recordings in vehicle-(Con) and LPS-treated rats 24 h post induction (mean ± SEM; n = 5 animals/group; two-sided Mann-Whitney test; *p ≤ 0.05, **p ≤ 0.01).

There was a non-significant trend towards a hemodynamic response with a slight increase in heart rate and a decrease in systolic arterial pressure. In contrast, CBF measured 24 h after induction of sepsis was significantly reduced (two-tailed Mann-Whitney test; p = 0.031, Figure 1). Generalized EEG activity tended to be reduced (increase of delta activity, decrease of activity in other frequency bands and a decrease in the main EEG frequency), but these changes did not reach the level of statistical significance. However, further analysis of single activity bands revealed that alpha activity was significantly reduced (two-tailed Mann-Whitney test, p = 0.044). Nonparametric correlation analysis yielded a significant correlation of cortical blood flow changes and main EEG frequency (Spearman's rho = 0.304, p = 0.044).

### Brain region dependent effect of LPS on glucose uptake

**Figure 2:**
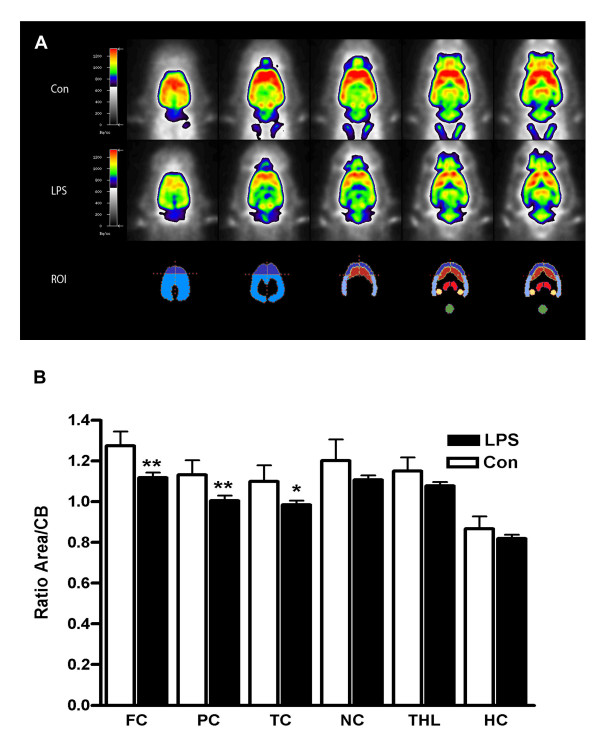
**Reduction of cerebral glucose uptake in septic encephalopathy**. Shown are five representative transversal [^18^F]FDG-PET brain slices of rats treated with vehicle (Con, first row) or bacterial lipopolysaccharide (LPS, second row) at 24 h. Corresponding region-of-interest (ROI) masks are displayed below. B, Quantification of [^18^F]FDG uptake (relative to cerebellar (CB) ROI) in vehicle-treated wild-type (Con) and LPS-treated mice (LPS) at 24 h. Significant differences were detected in frontal cortex (FC), parietal cortex (PC) and temporal cortex (TC). In contrast, evaluation of the caudate nucleus (NC), thalamus (THL) and hippocampus (HC) did not yield significant results (mean ± SEM; n = 5 animals/group; Student's t test; *p ≤ 0.05, **p ≤ 0.01).

In order to correlate the observed sepsis-induced changes in CBF and EEG to brain glucose utilization, a marker of neuronal activity, we assessed cerebral glucose uptake by 18F-fluordeoxyglucose positron emission tomography ([18F]FDG-PET) using a small animal scanner. Twenty-four h after application of LPS, brain glucose uptake was reduced in all neocortical areas in the LPS-treated group compared to the vehicle-treated group (Figure 2), but not in caudate nucleus, thalamus and hippocampus, suggesting differences in regional vulnerability of different brain areas in the model used.

### Sepsis induces microglial activation and neuronal cell loss

**Figure 3:**
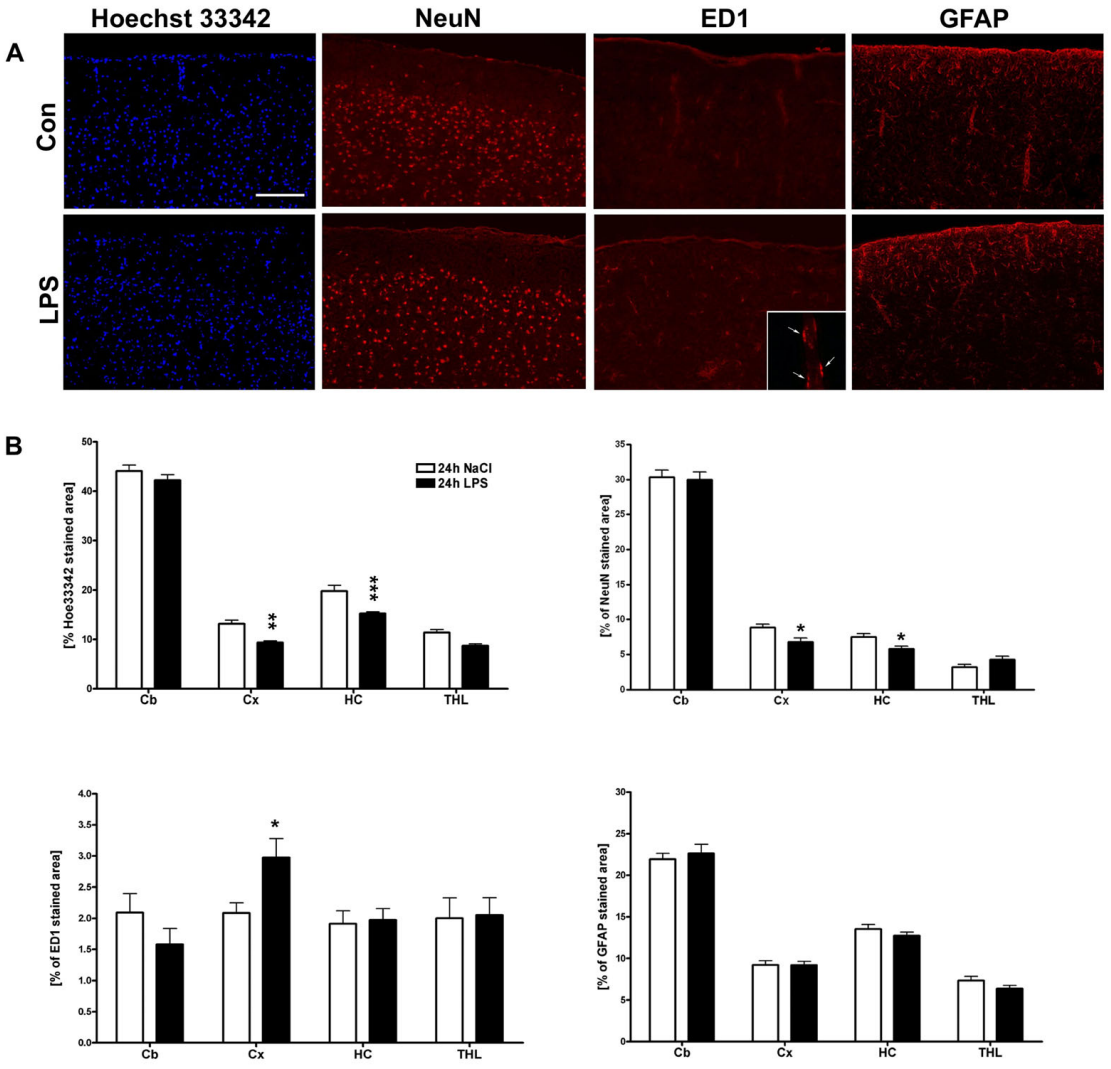
**Histological analysis of regional LPS induced changes of cell number, micro- and astroglial activation**. A, cell number detection by Hoechst 33342 staining and detection of microglial (ED1), neuronal (NeuN), and astroglial (GFAP) immunoreactivity in the cortex, 24 h after intraperitoneal application of LPS (LPS) or vehicle (Con). Results are given as percentage of immunopositive area. The inset shows a 100× magnification of ED1 positive microglial cells located along a cerebral blood vessel. (mean ± SEM; n = 5 animals/group; Student's t test; *p ≤ 0.05, **p ≤ 0.01). B, Quantitative analysis of Hoechst 33342 staining, ED1, NeuN, and GFAP immunostaining in the cortex (Cx), cerebellum (Cb), thalamus (THL), and hippocampus (HC).

Histological and immunohistochemical procedures showed that the observed hemodynamic, electrophysiological and metabolic changes in the brain were paralleled by cell loss in the cortex and hippocampus (Figure 3A and 3B). Additionally, staining for the neuronal marker NeuN revealed a reduction in the cortical layers and the hippocampus (Figure 3A and 3B). ED1-reactive microglial cells were found to be increased in LPS-treated animals, mostly located in close vicinity to brain blood vessels (Figure 3A and 3B), but also found with round to oval appearance scattered throughout the brain parenchyma (data not shown). We were not able to detect changes in GFAP immunoreactivity within 24 h after LPS administration (Figure 3A and 3B).

### LPS-induced changes of inflammatory markers

**Figure 4:**
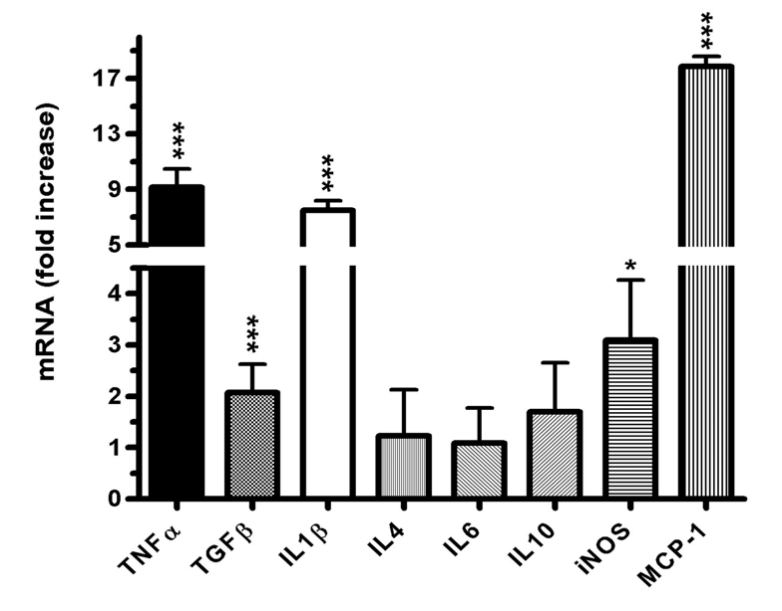
**Reduction of cerebral glucose uptake in septic encephalopathy**. Inflammatory gene transcription was studied using cDNA generated from whole brain lysates 24 h after intraperitoneal administration of either LPS or vehicle. Induction of the indicated pro- and anti-inflammatory cytokines was measured by real-time RT-PCR with RNA samples prepared from vehicle treated controls and LPS treated rats. Triplicate determination was performed for each mRNA sample. Gene-expression levels were normalized with GAPDH for each mRNA preparation, and the n-fold increase in LPS treated animals was calculated by comparison with the result obtained in untreated rats. Results are the mean ± (+SEM) of five animals per group. (Student's t test; *p ≤ 0.05, ***p ≤ 0.001).

RT-PCR of whole brain lysates showed that intraperitoneal injection of LPS caused an upregulation of various pro- and anti-inflammatory mediators within the brain, with the chemokine monocyte chemoattractant protein 1 (MCP-1) showing the strongest response, followed by the cytokines interleukin 1 beta (Il-1β), transforming growth factor beta (TGF-β) and tumor necrosis factor alpha (TNF-α, Figure 4). In addition inducible nitric oxide synthase (iNOS) mRNA levels were significantly increased (Figure 4). Several other cytokine levels, including interleukin 4, interleukin 6 and interleukin 10 were unchanged (Figure 4).

## Discussion

Sepsis-induced brain dysfunction receives increasing attention since it directly causes brain damage [9,10] and correlates well with morbidity and mortality rates of systemic septicemia [2]. Despite this fact, the established treatment protocols for patients suffering from sepsis or septic shock lack a specific neuroprotective approach, and the therapeutic strategy mainly focuses on antimicrobial drugs and stabilization of cardiovascular parameters. A prerequisite for the development of improved treatment approaches is the availability of appropriate animal models that use tools and techniques relevant to the clinical setting. In this study we aimed to investigate the mutual relation of CBF changes, EEG and brain metabolism in experimentally induced sepsis in rats. We correlated our findings to inflammatory gene transcription and histological analysis of neuronal loss as well as micro- and astroglial activation.

Induction of experimental sepsis by LPS did not significantly alter systolic blood pressure, heart rate or the calculated shock index at 24 h. Thus, LPS application did not cause a profound septic shock syndrome but rather resulted in slight changes reminiscent of a hyperdynamic circulatory status which can be observed in early stages of sepsis, where a marked peripheral vasodilatation is offset by a substantial increase in cardiac output resulting in little or no change of mean arterial blood pressure [11]. Of note, a mean arterial blood pressure within normal limits may resemble more closely the situation of patients who are treated with vasopressants if septic shock is present. Since the lower limit of cerebral autoregulation in rats is around 50 mmHg [12,13], it seems unlikely that the CBF reduction found in the present study is caused by systemic cardiovascular changes, but rather results from impaired cerebral microcirculation as recently reported in a similar model [14].

During human sepsis, EEG changes are common and the degree of EEG abnormalities is associated with the clinical severity and the prognosis of SE [5]. Although EEG changes found in septic rats were not as pronounced as those described in septic patients [5], our study yielded comparable results, revealing a generalized slowing of overall EEG activity and a significant decrease of alpha activity. These findings confirm previous observations that LPS-induced sepsis in rats reduced EEG activity in frequency bands ≥ 8 Hz [15] up to 12 h after LPS administration. In the latter study, a significant increase for frequencies between 2 and 4 Hz was only observed within the first 6 h of the experiment, as in our study where no significant changes of low frequency bands were detected at 24 h. Interestingly, both serotype and LPS concentration of the latter study were different to our experimental protocol, suggesting that the observed effects on EEG activity are independent from these factors. Paralleling the LPS-induced EEG changes, microPET *in vivo *analysis of cerebral glucose uptake, a metabolic process that has been linked to neuronal activity in rodents [16], was significantly reduced in all cortical areas examined. Explorative data analysis showed a significant correlation of slowing of the EEG activity and the decrease of regional CBF found in septic animals. Reduction of regional CBF might therefore, at least in part, be causative for the observed EEG-changes considered to reflect SE. Alternatively, the induction of sepsis may have caused substantial brain dysfunction through both, systemic release [17] as well as local generation of inflammatory molecules by peripheral immune cells or locally activated microglia. Such an activation of microglial cells and astrocytes, which both can serve as major source of inflammatory molecules has been well documented in rodent models of SE [18] and brains of septic patients [9]. In this study, we found that microglial activation in the cortex was associated with a significant increase of inflammatory gene transcription of Il-1β, TNFα, TGF-β, iNOS and MCP-1. In addition, we observed a reduction of total and neuronal cell number in the cortex and hippocampus, even so we can not exclude that this may be caused by swelling of the brain. Of note, both cytokines, TNF-α and Il-1β, have been reported to affect neuronal function [4,19,20] or survival [21,22]. Similarly, iNOS-derived NO can down regulate neuronal activity [23,24], and it has been shown that neuronal viability is remarkably sensitive to sustained iNOS dependent NO generation [25-27]. Thus, inflammatory molecules in concert with inflammation-triggered NO generation may directly impair neuronal function and cause neurodegeneration during SE. Neuronal cell death and a reduction of neuronal activity – as evidenced by reduced cerebral glucose utilization and EEG slowing, in turn, may negatively regulate the local CBF since the latter is directly coupled to the activity of the neighboring neurons [28,29]. It is therefore likely that during SE, cerebral microcirculatory failure, systemic and local inflammation and neuronal activity mutually influence and promote each other.

## Conclusion

Together, the present study highlights the complexity of these changes and the early impairment of neuronal metabolism and activity during SE.

Recent data suggest that sepsis causes long-term behavioral changes in rodents [30,31], and minor cognitive impairment might well be present in human sepsis survivors [3]. Since our model mimics key aspects of human SE, it might be used to develop brain specific therapeutic strategies for patients suffering from sepsis

## Competing interests

The authors declare that they have no competing interests.

## Authors' contributions

AS carried out most of the EEG and cerebral CBF experiments and generated a first draft of the manuscript. SH carried out the small animal PET experiments. FM participated in the EEG measurements. MW conducted the immunohistological analysis. SP carried out the qPCR analysis. TO participated in the EEG and CBF measurements. MS oversaw the small animal PET and interpreted the data. MPK helped to draft the manuscript and participated in the coordination. TG helped to draft the manuscript. MTH conceived of the study, and participated in its design and coordination and helped to draft the manuscript. All authors read and approved the final manuscript.

## References

[B1] Pine RW, Wertz MJ, Lennard ES, Dellinger EP, Carrico CJ, Minshew BH (1983). Determinants of Organ Malfunction Or Death in Patients with Intra-Abdominal Sepsis – A Discriminant-Analysis. Archives of Surgery.

[B2] Sprung CL, Peduzzi PN, Shatney CH, Schein RMH, Wilson MF, Sheagren JN (1990). Impact of Encephalopathy on Mortality in the Sepsis Syndrome. Critical Care Medicine.

[B3] Wilson JX, Young GB (2003). Progress in clinical neurosciences: Sepsis-associated encephalopathy: Evolving concepts. Canadian Journal of Neurological Sciences.

[B4] Pickering M, Cumiskey D, O'Connor JJ (2005). Actions of TNF-alpha on glutamatergic synaptic transmission in the central nervous system. Experimental Physiology.

[B5] Young GB, Bolton CF, Archibald YM, Austin TW, Wells GA (1992). The Electroencephalogram in Sepsis-Associated Encephalopathy. Journal of Clinical Neurophysiology.

[B6] Soehle M, Heimann A, Kempski O (2001). On the number of measurement sites required to assess regional cerebral blood flow by laser-Doppler scanning during cerebral ischemia and reperfusion. Journal of Neuroscience Methods.

[B7] Schafers KP, Reader AJ, Kriens M, Knoess C, Schober O, Schafers M (2005). Performance evaluation of the 32-module quadHIDAC small-animal PET scanner. Journal of Nuclear Medicine.

[B8] Boashash B (1992). Estimating and Interpreting the Instantaneous Frequency of A Signal .1. Fundamentals. Proceedings of the Ieee.

[B9] Sharshar T, Annane D, de la Grandmaison GL, Brouland JP, Hopkinson NS, Gray F (2004). The neuropathology of septic shock. Brain Pathology.

[B10] Nguyen DN, Spapen H, Su FH, Schiettecatte J, Shi L, Hachimi-Idrissi S (2006). Elevated serum levels of S-1000 protein and neuron-specific enolase are associated with brain injury in patients with severe sepsis and septic shock. Critical Care Medicine.

[B11] Parrillo JE (1993). Mechanisms of Disease – Pathogenetic Mechanisms of Septic Shock. New England Journal of Medicine.

[B12] Tonnesen J, Pryds A, Larsen EH, Paulson OB, Hauerberg J, Knudsen GM (2005). Laser Doppler flowmetry is valid for measurement of cerebral blood flow autoregulation lower limit in rats. Experimental Physiology.

[B13] Rosengarten B, Hecht M, Kaps M (2006). Carotid compression: Investigation of cerebral autoregulative reserve in rats. Journal of Neuroscience Methods.

[B14] Rosengarten B, Hecht M, Auch D, Ghofrani HA, Schermuly RT, Grimminger F (2007). Microcirculatory dysfunction in the brain precedes changes in evoked potentials in endotoxin-induced sepsis syndrome in rats. Cerebrovascular Diseases.

[B15] Lancel M, Mathias S, Schiffelholz T, Behl C, Holsboer F (1997). Soluble tumor necrosis factor receptor (p75) does not attenuate the sleep changes induced by lipopolysaccharide in the rat during the dark period. Brain Research.

[B16] Kornblum HI, Araujo DM, Annala AJ, Tatsukawa KJ, Phelps ME, Cherry SR (2000). In vivo imaging of neuronal activation and plasticity in the rat brain by high resolution positron emission tomography (microPET). Nature Biotechnology.

[B17] O'Dwyer MJ, Mankan AK, Stordeur P, O'Connell B, Duggan E, White M (2006). The occurrence of severe sepsis and septic shock are related to distinct patterns of cytokine gene expression. Shock.

[B18] Semmler A, Okulla T, Sastre M, Dumitrescu-Ozimek L, Heneka MT (2005). Systemic inflammation induces apoptosis with variable vulnerability of different brain regions. Journal of Chemical Neuroanatomy.

[B19] Tancredi V, Darcangelo G, Grassi F, Tarroni P, Palmieri G, Santoni A (1992). Tumor-Necrosis-Factor Alters Synaptic Transmission in Rat Hippocampal Slices. Neuroscience Letters.

[B20] Kelly A, Lynch A, Vereker E, Nolan Y, Queeman P, Whittaker E (2001). The anti-inflammatory cytokine, interleukin (IL)-10, blocks the inhibitory effect of IL-1 beta on long term potentiation – A role for JNK. Journal of Biological Chemistry.

[B21] de Bock F, Derijard B, Dornand J, Bockaert J, Rondouin G (1998). The neuronal death induced by endotoxic shock but not that induced by excitatory amino acids requires TNF-alpha. European Journal of Neuroscience.

[B22] Venters HD, Dantzer R, Kelley KW (2000). A new concept in neurodegeneration: TNF alpha is a silencer of survival signals. Trends in Neurosciences.

[B23] Mori K, Togashi H, Ueno K, Matsumoto M, Yoshioka M (2001). Aminoguanidine prevented the impairment of learning behavior and hippocampal long-term potentiation following transient cerebral ischemia. Behavioural Brain Research.

[B24] Wang QW, Rowan MJ, Anwyl R (2004). beta-amyloid-mediated inhibition of NMDA receptor-dependent long-term potentiation induction involves activation of microglia and stimulation of inducible nitric oxide synthase and superoxide. Journal of Neuroscience.

[B25] Boje KM, Arora PK (1992). Microglial-Produced Nitric-Oxide and Reactive Nitrogen-Oxides Mediate Neuronal Cell-Death. Brain Research.

[B26] Leist M, Fava E, Montecucco C, Nicotera P (1997). Peroxynitrite and nitric oxide donors induce neuronal apoptosis by eliciting autocrine excitotoxicity. European Journal of Neuroscience.

[B27] Heneka MT, Loschmann PA, Gleichmann M, Weller M, Schulz JB, Wullner U (1998). Induction of nitric oxide synthase and nitric oxide-mediated apoptosis in neuronal PC12 cells after stimulation with tumor necrosis factor-alpha lipopolysaccharide. Journal of Neurochemistry.

[B28] Ances BM (2004). Coupling of changes in cerebral blood flow with neural activity: What must initially dip must come back up. J Cereb Blood Flow Metab.

[B29] Chaigneau E, Tiret P, Lecoq J, Ducros M, Knopfel T, Charpak S (2007). The relationship between blood flow and neuronal activity in the rodent olfactory bulb. Journal of Neuroscience.

[B30] Barichello T, Martins WR, Reinke A, Feier G, Rifter C, Quevedo J (2005). Cognitive impairment in sepsis survivors from cecal ligation and perforation. Critical Care Medicine.

[B31] Semmler A, Frisch C, Debeir T, Ramanathan M, Okulla T, Klockgether T (2007). Long-term cognitive impairment, neuronal loss and reduced cortical cholinergic innervation after recovery from sepsis in a rodent model. Experimental Neurology.

